# Improving Control of Tuberculosis in Low-Burden Countries: Insights from Mathematical Modeling

**DOI:** 10.3389/fmicb.2016.00394

**Published:** 2016-05-03

**Authors:** Peter J. White, Ibrahim Abubakar

**Affiliations:** ^1^MRC Centre for Outbreak Analysis and Modelling and NIHR Health Protection Research Unit in Modelling Methodology, Imperial College London School of Public HealthLondon, UK; ^2^Modelling and Economics Unit, Centre for Infectious Disease Surveillance and Control, Public Health EnglandLondon, UK; ^3^TB Section, Respiratory Diseases Department, Centre for Infectious Disease Surveillance and Control, Public Health EnglandLondon, UK; ^4^Research Department of Infection and Population Health, University College LondonLondon, UK; ^5^MRC Clinical Trials Unit, University College LondonLondon, UK

**Keywords:** MDR-TB, transmission, latent TB infection, screening, migrants, health systems, health economics, cost-effectiveness

## Abstract

Tuberculosis control and elimination remains a challenge for public health even in low-burden countries. New technology and novel approaches to case-finding, diagnosis, and treatment are causes for optimism but they need to be used cost-effectively. This in turn requires improved understanding of the epidemiology of TB and analysis of the effectiveness and cost-effectiveness of different interventions. We describe the contribution that mathematical modeling can make to understanding epidemiology and control of TB in different groups, guiding improved approaches to public health interventions. We emphasize that modeling is not a substitute for collecting data but rather is *complementary* to empirical research, helping determine what are the key questions to address to maximize the public-health impact of research, helping to plan studies, and making maximal use of available data, particularly from surveillance, and observational studies. We provide examples of how modeling and related empirical research inform policy and discuss how a combination of these approaches can be used to address current questions of key importance, including use of whole-genome sequencing, screening and treatment for latent infection, and combating drug resistance.

## Introduction

Tuberculosis remains a public health challenge, even in low-burden countries, such as the USA (Hill et al., 2012) and European countries (Abubakar et al., 2012a; Lönnroth et al., 2015). TB epidemiology is complicated, with multiple interacting risk factors determining the burden of disease in different population groups (de Vries et al., 2014). Generally, most cases of disease in many low-burden countries arise from imported latent TB infection (LTBI), but homeless and other deprived groups can have transmission rates as high as some high-burden countries (e.g., White et al., 2011). With limited budgets and competing health priorities there are important questions regarding the best tools and practical approaches, tailored to patients’ needs, for diagnosis and treatment of latent and active TB (**Table 1**).

**Table 1:** Current key questions for research in to TB control in low-burden countries.

Natural history of infection and transmission patterns in low-burden countries
• How does the natural history of TB, including rates of progression from LTBI to active disease, vary amongst population groups and with different risk factors?
• How much TB disease in migrants from high-burden countries is due to LTBI at the time of migration, acquisition whilst in the low-burden country, and acquisition whilst visiting the country of origin or receiving visitors from that country? (This informs on how much disease is potentially preventable by reducing transmission in the low-burden country.)
• How much transmission occurs in households, workplaces, etc?
• What is the impact and cost-effectiveness of whole-genome sequencing on identifying drug resistance patterns (for individual patient care) and transmission clusters (to inform public health responses)?
• What is the impact of LTBI testing and treatment on disease and transmission in different groups?
• What is the (cost-)effectiveness of contact investigation in different groups?
MDR-TB
• What are the transmission patterns of MDR-TB?
• What are the most effective therapeutic options for MDR-TB, including optimizing the trade-off of providing access to new drugs whilst protecting those drugs from emergence of resistance?
Provision of care
• What is the cost-effectiveness of LTBI screening of different migrant groups, delivered in different ways?
• What are the costs of diagnosis and treatment for LTBI and active TB provided to different patient groups in different health care settings and what are the most cost-effective ways of providing diagnosis and care?
• What are the most (cost-) effective packages of care for different patient groups?
• What is the (cost-) effectiveness of combining active case finding for TB with screening for other infections (e.g., HCV, HIV) in particular risk groups, such as homeless persons and prisoners?
• What is the effectiveness and cost-effectiveness of different approaches to case-management to promote adherence?
Vaccination
• What is the (cost-)effectiveness of BCG vaccination in different groups?
• What is the minimum effectiveness of novel vaccines required for their use to be cost-effective?


Modeling provided insights into TB natural history, epidemiology, and control for decades (White and Garnett, 2010 extensively reviews modeling of natural history; here we focus on other uses). Whilst models can be used to examine “what if” scenarios (Abu-Raddad et al., 2009; Garnett et al., 2011; Hill et al., 2012), calculating the expected impact of interventions that have not (yet) been implemented, modeling is not a substitute for empirical study but rather a tool to analyze empirical data. Indeed, modeling is used to synthesize evidence from multiple sources (Drobniewski et al., 2015 reports multiple studies, including modeling). Complementary analytical approaches include trials; observational studies (Jit et al., 2011; Pareek et al., 2011a, 2013); mapping of clinical pathways (Drobniewski et al., 2015; Green et al., in preparation) and surveys of clinical practice (Pareek et al., 2011b); analysis of aggregate data from surveillance (Drobniewski et al., 2015) and screening programs (Aldridge et al., 2016); analysis of patient records, including database linkage to link laboratory and surveillance data, and to link risk factors to outcomes (Baussano et al., 2006; Anderson et al., 2014; Stagg et al., 2015, 2016; Aldridge et al., 2016; Aldridge et al., in preparation); and systematic reviews (Tiemersma et al., 2011; Aldridge et al., 2014; Stagg et al., 2014).

In this paper we concentrate on modeling of TB to inform public health in high-income, low-burden countries.

## Infectious-Disease Transmission Dynamics and Cost-Effectiveness Calculation

Infectious diseases are transmitted from one host to another, and control requires interrupting this process, which involves interaction between individual-level and population-level processes, known as *transmission dynamics* (Abubakar, 2016; Cohen and White, 2016; White, in press). The *incidence* of infection – the rate new infections arise in the population per unit time – depends on the *prevalence* of infectious individuals in the population (the proportion of the population that is infectious) at the time. Of course, prevalence depends upon the rate new infections have been arising (and rates of recovery and death), so prevalence depends upon incidence. Therefore for infectious diseases there is a dynamic feedback loop, with incidence depending upon prevalence and prevalence depending upon incidence.

Transmission dynamics have important consequences for intervention effectiveness and cost-effectiveness [hereafter “(cost-)effectiveness”], because interventions such as vaccination and treatment of infection can benefit not just individuals receiving the intervention but can also benefit the population by averting transmission, improving health and reducing the number of infections needing to be treated, saving money (Cohen and White, 2016; Jit and White, 2016). Indeed, population-level benefits can be the major benefit of intervention and can even result in interventions being cost-saving. If a vaccine with sufficient efficacy and duration of protection is available then very large reductions in disease burden can be achieved, if population coverage is sufficient to achieve ‘herd immunity’; indeed, for some infections elimination is possible.

Economic analysis of infectious-disease interventions requires specialized *transmission-dynamic* mathematical models, integrating transmission dynamics, and health economics (Cohen and White, 2016; Jit and White, 2016; White, in press). Standard (“*static*”) health-economic models (e.g., decision trees, Markov models), developed for non-infectious diseases, are simpler because treating or vaccinating a patient does not affect incidence of disease in others (Jit and White, 2016). Whilst it can sometimes be acceptable to use static models for infectious diseases, e.g., in settings where transmission is rare (Jit and Brisson, 2011) or where an intervention is cost-effective without considering transmission-dynamic benefits it is preferable not to do so. Even in low-burden countries, where most TB cases are due to imported infection, transmission is still a concern.

## Uses of Mathematical Modeling to Address Issues in Tuberculosis Public Health

### Extrapolating Long-Term Outcomes Using Proxy Measures from Trials

In trials, there is usually not time to follow patients to their ultimate end-point: the individual benefit of being cured of active tuberculosis is typically years or even decades of life gained by those patients who would otherwise have died, and reduced morbidity in all patients. Therefore, determining the full health benefit requires modeling to calculate life-years gained in a treated patient cohort, accounting for their life expectancy if treatment averts death from TB. In health-economic analysis life-years gained are usually used to calculate Quality-Adjusted Life-Years (QALYs) gained, which incorporate reducing morbidity – increasing quality of life – as well as by reducing mortality, increasing duration of life (Jit and White, 2016). [Disability-Adjusted Life-Years (DALYs) are an alternative, measuring health detriment rather than gain]. Modeling of LTBI treatment (White and Jit, 2015), taking evidence of treatment efficacy from trials and calculating QALYs gained by the use of different regimens in different age-groups, informed guidance from England’s National Institute for Health and Care Excellence (NICE) (Hoppe et al., 2016).

**FIGURE 1:**
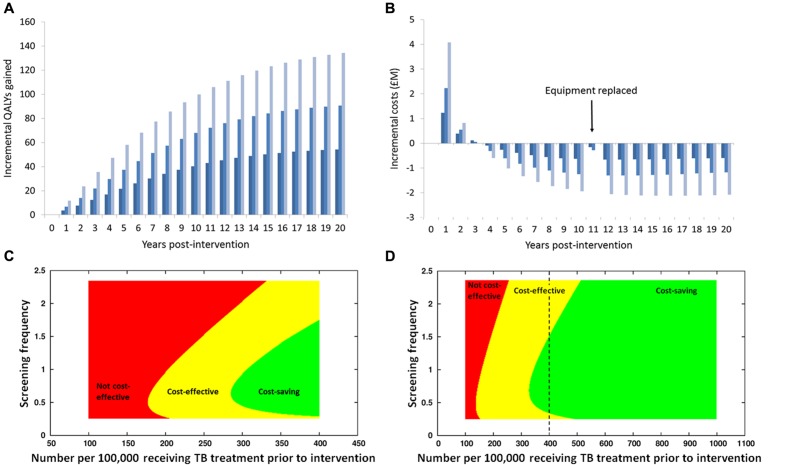
**Results of an economic analysis of screening homeless persons and prisoners for active TB using a mobile X-ray unit, using the model in White et al. (2011).** **(A)** Health benefits (in terms of Quality-Adjusted Life Years, QALYs) gained in each year, with discounting at 3.5% per annum, with different colors representing different frequencies of screening (dark blue: lowest rate; light blue: highest rate). Notice how the health benefits increase over time, as reductions over time in prevalence of infectious individuals reduce rates of new infections occurring. **(B)** Annual net costs: initially, there are costs incurred in purchasing equipment and increased treatment costs, due to infections being found earlier than otherwise, including some individuals being found and treated who would otherwise have died of tuberculosis. In later years the net cost is negative, meaning that the intervention saves money, because the cost of the intervention is less than the money saved due to the reduction in cases occurring and requiring treatment. Note that the different temporal patterns in health benefits and costs means that the time-horizon considered has a major impact on the calculated cost-effectiveness: in the first 2–3 years there is a net cost to the intervention and the health benefits are relatively modest, but over a longer period the intervention becomes cost-saving and the health benefits are large. **(C,D)** Examination of how the cost-effectiveness of screening **(C)** homeless persons and **(D)** prisoners for active TB using a mobile X-ray unit depends upon the prevalence of disease and the frequency of screening. On the horizontal axis is the prevalence prior to the introduction of screening; note that the range of values for homeless persons is greater than for prisoners (reflecting empirical observations); the dashed vertical line on the graph for the homeless indicates the extent of the range considered for prisoners. On the vertical axis is the screening frequency, with the value 1 corresponding to the ‘middle’ frequency **(A,B)**. At high prevalence screening is cost-saving as well as beneficial to health (green area), due to a high yield of screening and a large number of infections averted due to a high transmission rate in the absence of intervention, which means that averted treatment costs exceed the cost of the intervention. At intermediate prevalence, screening is not cost-saving but is still cost-effective (there is a net cost to the intervention but the health benefits are sufficient for the cost per QALY gained to be below the cost-effectiveness threshold), whereas at lower prevalence, screening is not cost-effective (the health benefits are in sufficient for the intervention to the considered worth the cost). Note that there is an optimal frequency of screening. Due to the curved boundary of the area corresponding to screening being cost-saving, for some intermediate values of prevalence screening at a low frequency is cost-effective but increasing the screening frequency makes the intervention cost-saving – due to a greater reduction in transmission – whilst increasing to even higher frequency makes screening no longer cost-saving, and potentially not cost-effective: this is due to “diminishing returns” if the screening frequency is increased to excessively high levels.

Transmission-dynamic effects mean benefits of interventions can continue – and indeed *increase* – in to the future and even beyond the lifetimes of the individuals receiving the intervention and their contemporaries in the wider population (Jit and White, 2016). This is particularly true for TB, with its long incubation period, making it important to consider the long term, as costs of interventions are typically greater in the early stages with the benefits accruing over time (**Figure 1**; White et al., 2011).

### Analyzing Data from Observational Studies When a Trial Is Not Feasible

Often trials are not feasible, so observational data have to be analyzed to assess intervention effectiveness without having patients recruited to a control arm for comparison. Modeling can calculate a counterfactual – i.e., what would have occurred in the absence of the intervention – to compare with what was observed (Garnett et al., 2011). This approach assessed the cost-effectiveness of the London TB Find and Treat service, which used a mobile X-ray unit to screen homeless persons and prisoners for active TB and provided case-management support (Jit et al., 2011), with a synthetic control group constructed from surveillance records of individuals who had received standard case-finding and care. This analysis did not use a transmission-dynamic model, due to time constraints, with urgent analysis required to inform policy: as the intervention was cost-effective without considering benefits of averting transmission one could be certain it was even more cost-effective than calculated.

Screening of immigrants to low-burden countries from higher-burden countries (Pareek et al., 2011b; Aldridge et al., 2014) provides an example of modeling answering “what-if” questions by examining a range of scenarios not feasible to test in a trial – specifically, the cost-effectiveness of screening and which countries should be included in the program. Recently, Aldridge et al. (2014) reviewed reported yields of active TB from pre-entry screening programs, and analyzed UK data in detail (Aldridge et al., 2016); these studies can be used to inform health-economic modeling. Pareek et al. (2011a) measured LTBI prevalence in immigrants entering the UK from a range of countries and modeled the cost-effectiveness of different “screening thresholds” using WHO-estimated TB burden in countries of origin. Not screening migrants from the Indian sub-continent, following guidance at the time, was missing 70% of imported LTBI. Further analysis (Pareek et al., 2013) compared different screening strategies, with and without chest X-ray and tuberculin skin test (TST) and two interferon-gamma release assays (IGRAs), and found that single-step screening with the QuantiFERON Gold in-Tube IGRA was most cost-effective.

Recent analysis informing NICE guidance on LTBI treatment examined the cost-effectiveness of different regimens and treating different age-groups: whilst older individuals will have fewer years at risk of progression to active TB, and fewer years of life lost if they develop active TB and die of it, and are at greater risk of adverse events from LTBI treatment, they also have a greater risk of dying from active TB if they develop it, and modeling is required to determine the overall outcome of this combination of factors (White and Jit, 2015). A key parameter is the risk of progression to active disease; recent analysis of migrants to the UK provides updated estimates (Aldridge et al., in preparation).

### Assessing Interventions at Full Scale

An important consequence of transmission dynamics is that the scale of intervention affects its effectiveness: a major benefit of a successful intervention is averting infections, but small-scale trials are unlikely to reduce transmission at the population level even if the intervention is successful. Large-scale cluster randomized control trials (cRCTs) are more likely to reduce incidence detectably but any reduction is still likely to be proportionately less than a full-scale intervention and it is usually not feasible to conduct cRCTs at a scale that can measure full benefits. When a public health intervention has been implemented at scale then modeling can be used to calculate a counterfactual to compare with observational data from surveillance systems (Hallett et al., 2007).

Hill et al. (2012) used modeling to assess whether the USA is likely to eliminate TB by 2100 with then-current approaches and concluded it was unlikely, and Davidow et al. (2015) concluded that expanding LTBI testing and treatment of migrants would be required, along with improving TB control internationally. Dye et al. (2013) modeled elimination in South Africa, India, China, and the USA, and concluded that implementation of the WHO Stop TB Strategy, is required, along with new technology “including biomarkers of TB risk, diagnostics, drugs, and vaccines”.

### Examining Combinations of Interventions and Accounting for Epidemiological Context

When different combinations of interventions are to be evaluated, it is usually not feasible to conduct trials measuring directly the effectiveness of each combination in comparison with all the other combinations (factorial designs are usually only practicable for two interventions, with four combinations). This is particularly the case for public health interventions where epidemiological context matters (e.g., heterogeneous disease burden in different locations or population groups), and the combinations of interventions and contexts need to be considered (e.g., targeting different interventions to different groups/locations). For example, the cost-effectiveness of screening homeless persons and prisoners for active TB using a mobile X-ray unit depends upon the prevalence of infection in the population and the frequency of screening (**Figure 1**; White et al., 2011). Combining interventions, there can be *synergy* or *redundancy* – the combination having greater or lesser impact than the sum of the individual interventions, respectively (Dodd et al., 2010). Synergy is most likely when the interventions target different aspects of the pathogen’s transmission process and life cycle and when the interventions are individually only moderately effective at most; combining such interventions could result in a ‘package’ that is effective and cost-effective. Dye et al. (2013) reported that targeting latent TB and active TB together can be synergistic.

### Health-Systems Modeling

With the advent of improved TB diagnostics, an important use of modeling is assessing the impact of incorporating them into the health system (Cobelens et al., 2012; Lin et al., 2012). Modeling has been used to assess the cost-effectiveness of introducing molecular testing in high-burden countries (Menzies et al., 2012) but assessments are also required for low-burden countries. Molecular tests for active TB and drug-resistance have lower sensitivity and/or specificity than microbiological culture, so have more-frequent false-negative and false-positive results, but are much faster than culture, so there are trade-offs to consider (Drobniewski et al., 2015). False-negative results delay treating some infections and increase the opportunity for transmission – although if molecular testing is used in addition to culture rather than replacing it then ultimately the infection would still be diagnosed as previously. False-positive results cause inappropriate treatment for TB (if the patient does not have the infection) and/or for Multidrug-Resistant TB (MDR-TB) (if the patient has TB but it is not MDR or does not have TB at all), incurring costs and health detriment (due to side-effects). These downsides need to be weighed against faster diagnosis and appropriate treatment where the test results are correct, resulting in improved prognosis, and reduced time that smear-positive patients may spend in isolation awaiting drug-susceptibility testing. Important factors are the proportion of patients tested who have TB and MDR-TB (which can vary amongst demographic groups); the sensitivity, specificity and cost of the molecular test; and the cost of patient isolation. In the UK, it is likely that adding molecular testing to diagnostic pathways would be beneficial to health and potentially cost-saving (Drobniewski et al., 2015). However, information on the details of health-system costs (e.g., durations of hospital stays; healthcare provided in general practice; staff time required for diagnostic testing, sample analysis, and patient care) is often limited (Drobniewski et al., 2015), and detailed studies of pathways is required (Green et al., in preparation). Additionally, there can be wide variation locally in clinical practice, as Pareek et al. (2011b) found for LTBI screening of immigrants, with activity generally inversely related to disease burden.

### Multidrug-Resistant TB

Controlling MDR-TB is a key concern in Europe, even in low-MDR-burden countries (Abubakar et al., 2012b; Drobniewski et al., 2015). Surveillance and modeling have important roles to play (Wells et al., 2013). However, whilst modeling has provided valuable insights (Cohen et al., 2009), it has been largely limited to examination of scenarios, due to a lack of detailed data making it difficult even to determine trends (Cohen et al., 2014).

Studying MDR-TB treatment, evolution, transmission, and control in low-MDR-burden countries is challenging due to relatively small numbers of cases (Drobniewski et al., 2015), although we do know that (e.g.) transmission is currently limited in England and tends to be concentrated in high-risk groups (Anderson et al., 2014). Studies in countries with higher MDR-TB burdens are therefore valuable, with central and eastern Europe being most relevant to lower-burden western Europe, considering the social context and health system structure (Stagg et al., 2015, 2016).

## Uncertainty

Modeling offers important insight into uncertainty, including quantifying how much arises from different causes (Cohen and White, 2016; Jit and White, 2016), which helps identify priorities for empirical research (Cohen et al., 2009). Modeling can identify important sources of uncertainty that may otherwise be overlooked, e.g., contact patterns that are important for rates of transmission of infection amongst and between population groups (Mossong et al., 2008).

There is uncertainty in TB’s natural history, not just in terms of values of parameters (“parametric uncertainty”, e.g., proportions of incident infections that are smear-negative vs. smear-positive, progression rates from latent infection to active disease, etc.) but also in terms of infection states which occur and which processes affect transitions between those states (“structural uncertainty”). Different formulations of mathematical models indicates the uncertainty in how TB natural history is best represented (White and Garnett, 2010), e.g., whether incident infections should bifurcate into slow- and fast-progressing infections or whether all infections should pass through a state when the risk of progression is high and then those who have not yet progressed enter a state where progression rates are lower, and whether exogenous reinfection promotes progression to active disease.

Whether uncertainty in parameter values (or model structure) leads to uncertainty for decision-making depends upon whether the probability density is concentrated on one side of the cost-effectiveness threshold or whether there are important proportions of the density on either side of the threshold.

## Conclusion

In low-burden countries TB control needs to be targeted appropriately for different patient groups. Targeting requires resources, so it needs to be efficient in finding and diagnosing at-risk persons. Assessing intervention cost-effectiveness requires transmission-dynamic modeling to determine numbers of infections averted, informed by data from empirical studies, e.g., of diagnostic tests in different settings (Drobniewski et al., 2015), and BCG vaccination (Abubakar et al., 2013).

There are important questions regarding TB’s natural history and epidemiology, which will affect the impact of interventions, including use of novel technologies such as new vaccines (**Table 1**). Whilst modeling can be used to gain insight into the underlying causal mechanisms giving rise to observed patterns, which can then inform control strategies, it is necessary to determine in detail what the patterns are, requiring detailed empirical study, particularly for MDR-TB strains.

Many interacting factors affect transmission in populations, including demographic and other risk factors, patterns of contact within and between groups having different work places, socializing in different venues, living in different settings (including homeless hostels), differing in their access to health services for diagnosis, and having different needs in being adherent to treatment. Migrants also differ in their risks of having acquired infection overseas vs acquiring it in a low-burden country post-migration. These different factors need to be quantified, to enable modeling ‘translate’ findings from the setting where a study was performed to other settings. Improved quantification of risk factors and rates of transmission is now possible, using improved laboratory methods, including whole-genome sequencing (e.g., Roetzer et al., 2013; Anderson et al., 2014; Walker et al., 2014), combined with high-quality epidemiological data – including contact tracing, and detailed documentation of risk factors and history of exposure – and mathematical modeling offers the prospect of important insights (Comas and Gagneux, 2009, 2011; Bryant et al., 2013; Roetzer et al., 2013; Croucher and Didelot, 2015; Theron et al., 2015). Population-based prevalence surveys (e.g., Miramontes et al., 2015) are also valuable.

Modeling has important roles in synthesizing data from multiple sources, setting research priorities by determining the key knowledge gaps causing uncertainty, designing and analyzing research studies and evaluating interventions using surveillance data. Finally, we emphasize that effective modeling requires multidisciplinary teams to ensure data are interpreted correctly and models are designed to address key questions of public health importance.

## Author Contributions

The paper was conceived by both authors. PW wrote the first draft, which was revised by both authors.

## Conflict of Interest Statement

PW has received research funding from Otsuka SA for a retrospective study of multidrug-resistant tuberculosis treatment in several eastern European countries. IA declares no competing interests.
